# Perceived Stress and risk of infection among Covid-19 frontline Healthcare workers

**DOI:** 10.1192/j.eurpsy.2022.1380

**Published:** 2022-09-01

**Authors:** A. Haddar, I. Sellami, A. Abbes, R. Masmoudi, H. Halweni, N. Derbel, K. Hammami, J. Masmoudi, M.L. Masmoudi, M. Hajjaji

**Affiliations:** 1 Hedi Chaker University Hospital of Sfax, Occupational Medecine, Sfax, Tunisie, Tunisia; 2 Hedi Chaker university hospital, Occupational Medicine, Sfax, Tunisia; 3 Hedi Chacker Hospital, Occupational Medicine, Sfax, Tunisia; 4 HEDI CHAKER hospital, Psychiatry Department, SFAX, Tunisia; 5 medecine university of Sfax, General Practice, Sfax, Tunisia; 6 HEDI CHAKER hospital, Department Of Occupational Medicine, SFAX, Tunisia; 7 CHU Hedi CHaker hospital Sfax Tunisia, Department Of Psychiatry (a), Sfax, Tunisia

**Keywords:** Covid-19, healthcare workers, Perceived stress

## Abstract

**Introduction:**

Working at Covid-19 frontline may threaten physical and mental health healthcare workers (HCWs). Assessing perceived Stress in HCWs is important to prevent serious mental illness

**Objectives:**

Assess the association between perceived stress and risk perception among healthcare workers working in the Covid-19 unit.

**Methods:**

We conducted a cross-sectional study among healthcare workers working in a COVID-19 unit between March and July 2021 through a self-administered questionnaire. The Perceived Stress Scale (PSS-10) was used to assess perceived stress. The perceived risk of getting infected was assessed by a 5-item Likert Scale. The perceived risk of family members’ infection was evaluated by a Scale from 0 to 10.

**Results:**

A total of 85 participants were included. The mean age was 31±6 years. About 87% of participants lived with their families. Seventy-six per cent of HCWs rated their health status greater than or equal to 8/10. A percentage of 18,8% of HCWs have been infected with the Covid-19. Our population consisted of 44.7% technicians and 24,7% nurses, and 80% of the participants reported direct contact with COVID-19 confirmed patients. The level of the perceived risk of getting infected was high to very high in 95% of the HCWs. The mean score of the perceived risk of family members contracting the virus was 5.7 and 27,1% rated it greater than or equal to 8. The PSS-10 showed moderate and high perceived stress in 82,3% and 7,1% of participants, respectively. Only 10,6% of HCWs presented low-stress perception.

**Conclusions:**

Frontline healthcare providers have high perceived stress and are at risk of mental health disorders

**Disclosure:**

No significant relationships.

